# Commentary on: Nomograms based on inflammatory biomarkers for predicting tumor grade and microvascular invasion in stage I/II hepatocellular carcinoma

**DOI:** 10.1042/BSR20190683

**Published:** 2019-10-15

**Authors:** Liang Xiao, Furong Zeng, Guangtong Deng

**Affiliations:** 1 General Surgery Department, Xiangya Hospital, Central South University, Changsha, China; 2 Xiangya Hospital, Central South University, Changsha, China

**Keywords:** HCC, microvascular invasion, nomogram

## Abstract

Some doubts were generated during the reading of nomograms based on inflammatory biomarkers for preoperatively predicting tumor grade and microvascular invasion in stage I/II hepatocellular carcinoma (HCC). We would like to highlight and discuss with authors. First, neutrophil-lymphocyte ratio (NLR) and derived NLR (dNLR) should not be entered into multivariate analysis simultaneously. Second, authors should clarify how the cutoffs of these variables including lymphocyte-monocyte ratio (LMR), dNLR, age and tumor size were set. We insist that the type of variables should be consistent when we carry out the analysis and establish the nomogram. Last, we have to point out that Li et al.’s (*Biosci. Rep.* (2018), **38**) study failed to validate nomograms using an independent dataset.

Dear editor,

We read with great interest in the recent paper regarding nomograms based on inflammatory biomarkers for preoperatively predicting tumor grade and microvascular invasion in stage I/II hepatocellular carcinoma (HCC) [1]. The nomogram for microvascular invasion plays a pivotal role in clinical decision-making. As we know, microvascular invasion is an extremely important independent risk factor for postoperative recurrence of HCC [2]. Microvascular invasion can not only help clinicians to develop therapeutic schedules after surgery, but also preoperatively guide surgeons on whether to perform liver transplantation beyond Milan Criteria, anatomical liver resection and widening of the surgical margin [3–6]. However, some doubts were generated during the process of reading that we would like to highlight and discuss with authors.

First of all, about neutrophil-lymphocyte ratio (NLR) and derived NLR (dNLR). We note that dNLR is composed of neutrophils count divided by white cells count minus neutrophils count. The latter, mixture of monocytes and lymphocytes, is broadly similar to the lymphocytes count. Therefore, dNLR is an alternative for NLR especially when we are not able to get access to lymphocytes count because usually neutrophils and white cells counts are available in clinical database [7]. What is more, studies show that dNLR has similar or inferior prognostic value to the NLR [7,8]. Therefore, it is recommended to choose either one of them to avoid an interaction effect when we analyze the data through multivariate logistic regression [9,10]. However, in Li et al.’s [1] study, NLR and dNLR are entered into multivariate analysis simultaneously. They concluded that not NLR but dNLR is the independent factor of microvascular invasion [1]. We feel that the conclusion of this study should be interpreted with some caution.

Second, about the binary variables in nomograms including lymphocyte-monocyte ratio (LMR), dNLR, age and tumor size. It is of note that these variables are analyzed as continuous variables through univariate and multivariate regression. However, these continuous variables were turned into binary variables in nomograms without explanation. Authors should clarify how the cutoffs of these variables were set. In my opinion, it is a wise choice to keep continuous variables in nomograms to avoid the massive information loss just like these papers [11,12]. We insist that the type of variables should be consistent when we carry out the analysis and establish the nomogram.

Last, we have to point out that Li et al.’s [1] study failed to validate nomograms using an independent dataset. The validation could help to avoid potential interobserver variability [13]. Also, the nomograms are based on the data from a single center. Thus, we could not conclude whether the nomograms work in other institutes.

In conclusion, the present study established practical nomograms for preoperatively predicting tumor grade and microvascular invasion. We hope that the authors can respond to our doubts and this will promote the predictive models.

## Abbreviations

dNLR: derived neutrophil-lymphocyte ratio
HCC: hepatocellular carcinoma
NLR: neutrophil-lymphocyte ratio
